# Interface Design of SnO_2_@PANI Nanotube With Enhanced Sensing Performance for Ammonia Detection at Room Temperature

**DOI:** 10.3389/fchem.2020.00383

**Published:** 2020-06-03

**Authors:** Anqiang Jia, Bitao Liu, Haiyan Liu, Qiufeng Li, Yingxia Yun

**Affiliations:** ^1^Department of Urban and Rural Planning, School of Architecture, Tianjin University, Tianjin, China; ^2^Research Institute for New Materials Technology, Chongqing University of Arts and Sciences, Chongqing, China; ^3^Institute of Urban and Rural Construction, College of Science, College of Animal Science and Technology, Hebei Agricultural University, Baoding, China

**Keywords:** ammonia sensor, room temperature, oxygen vacancies, PANI-T-SnO_2_, interface design

## Abstract

Gas sensors with excellent stability and a high response at room temperature has drawn a great deal of attention and demand for them is huge. Surface designs provide inspiration toward making more useful sensor devices. The facile electrospinning process and Ar plasma treatment are used to fabricate rich and stable oxygen vacancies that contain a core-shell structured SnO_2_ polyaniline (PANI) nanotube. It shows that the induced surface oxygen vacancies would accelerate the PANI shell to generate more protons, which can enhance its sensor responsibility through reacting with the target Ammonia (NH_3_) gas. It was also found that the obtained oxygen vacancies can be well-protected by the coated PANI shell, which enhance and stabilize the gas response. It shows that the room temperature for the gas response of NH_3_ can reach up to 35.3 at 100 ppm. Finally, its good stability is demonstrated by the response-recovery performances carried out over 3 months and multiple cycles. This work indicates that this well-designed PANI-coated plasma-treated SnO_2_ is a potential way to design ammonia gas sensors.

## Introduction

Ammonia (NH_3_), a colorless, strong, volatile gas, which can have a huge impact on the respiratory tract and eyes at a concentration below 50 ppm, has drawn much attention (Timmer et al., 2005; Li et al., 2016). It is generally known that human body's long-term allowable limit of NH_3_ in an indoor environment is lower than 25 ppm (Li et al., 2018). Thus, it is critical for environmental protection and human health to detect NH_3_ gas at room temperature. Oxide semiconductors have been extensively studied to detect dangerous and various toxic gases, especially in harsh environments (Cheng et al., 2017; Lupan et al., 2018). Dioxide (SnO_2_), which has a superior thermal stability (the melt point is 1,127°C), non-toxicity, and a low cost, has been proved to be an outstanding candidate for gas sensing (Wang et al., 2010; Das et al., 2014; Lee et al., 2015; Singkammo et al., 2015). Different morphologies of SnO_2_ have been synthesized for various gas detection, especially for the 1D nanostructured (Das et al., 2014; Lee et al., 2015; Singkammo et al., 2015). However, the optimized response is usually performed at a relatively high temperature of more than 200°C, which means that is not suitable for gas sensing at a room temperature (Das et al., 2014; Lee et al., 2015; Singkammo et al., 2015). Thus, it is still a challenge to reduce the working temperature.

Many attempts aimed at improving the properties of the gas sensor, like element doping (Wang et al., 2010), composites with RGO (Su and Yang, 2016; Chen et al., 2017) and MWCNT (Tyagi et al., 2017), and using a heterostructure design (Chen et al., 2015; Lee et al., 2015; Liu et al., 2016; Yuan et al., 2020), have been conducted. More importantly, inorganic and organic composites have drawn much attention for they can overcome the shortcomings of each other in gas sensor performance. Specifically, the conductive polymers can work effectively at room temperature, and among these, PANI has been considered a better NH_3_ sensor at room temperature (Shin et al., 2008; Wen et al., 2013; Li et al., 2016; Syrovy et al., 2016; Kumar et al., 2017; Bai et al., 2018; Liu B. T. et al., 2018; Nie et al., 2018; Tang et al., 2018, 2019; Santos et al., 2019). Several PANI and oxide semiconductors composites, like V_2_O_5_@PANI (Santos et al., 2019), CeO_2_@PANI (Liu C. H. et al., 2018), SiO_2_@PANI (Nie et al., 2018), SnO_2_@PANI (Bai et al., 2018), etc., have been reported as effective room temperature gas sensors. However, the recovery time for these PANI-based composites is usually long due to their hydrophilic surface. Additionally, PANI is a p type material that needs a fast electron speed to provide protons for the target gas effectively and provide a higher gas-sensitivity (Kumar et al., 2017). Thus, composites of PANI to an n-type oxide semiconductor material with an efficient electron speed could provide an opportunity to improve their gas-sensitive response through separating the accepted electrons (Wang et al., 2017; Chen et al., 2020a,b). Oxygen vacancies, an ideal electron acceptor, usually exist in many n-type oxide semiconductor materials, which can enhance the sensor properties and decrease the working temperature (Pacchioni, 2003; Trani et al., 2009). It has been reported that oxygen vacancies with rich oxide semiconductors, such as WO_3_ (Qin and Ye, 2016; Wang et al., 2017), W_18_O_49_ (Chen et al., 2020a), CeO_2_ (Soni et al., 2018) etc., would strengthen the interaction between vacancy-terminated surface and gas molecules remarkably, subsequently reducing the optimized work temperature and enhancing the sensitivity. Thus, developing oxygen vacancies with rich SnO_2_ through combining them with PANI should be an efficient way to obtain high sensitivity room temperature gas sensors.

As far as we know, there has been little research into the oxygen vacancies that contain SnO_2_-PANI for gas sensing at room temperature. Herein, in this work, we fabricated a nanotube core-shell structured SnO_2_-PANI nanotube with rich and stable oxygen vacancies to operate an NH_3_ sensor under room temperature. The oxygen vacancies were ingeniously introduced on the interface between the SnO_2_ nanotube and the PANI shell by Ar plasma treatment (Jia et al., 2019; Liu et al., 2019), which would promote the shell coated with PNAI to produce more protons for the target NH_3_gas and enhance its sensitivity.

## Experiment

**Figure 1:**
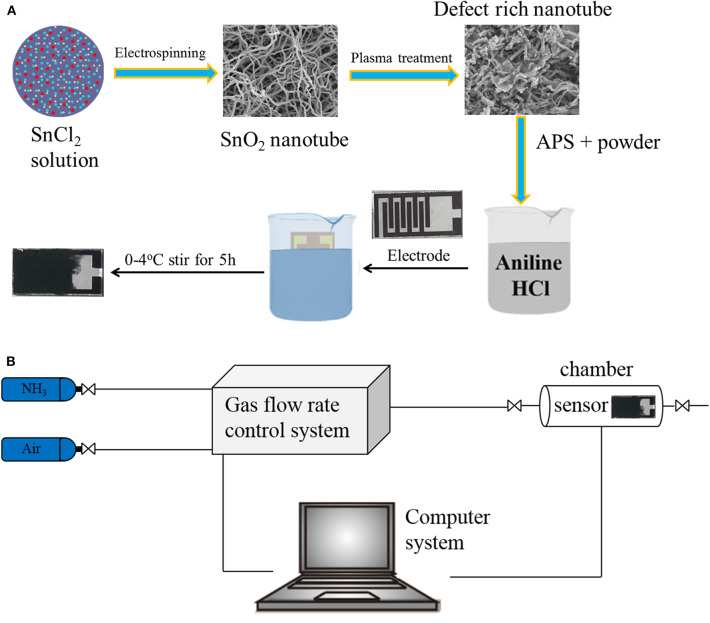
Schematic illustration of the deposition process of nanocomposite thin film **(A)**. Schematic diagram illustrating the gas-sensing test platform **(B)**.

The chemicals used in this experiment were of analytical purity; no further purification was required. The electrode was composed of glass substrate and gold. The schematic diagram of the samples synthesis process was shown in Figure 1.

### Synthesis of SnO_2_ Nanotube

Electrospinning was used to obtain the SnO_2_ nanotube according to ref (Wang et al., 2017). 0.4 g PVP and 2 g SnCl_4_·5H_2_O were dissolved in a mixture solution of 4.4 g DMF and ethanol, and this was then stirred for 6 h. A syringe with an inner diameter of 1.01 mm was used, the voltage was 18 kV, and the distance was 15 cm. The fibers were collected and dried for 12 h at 100°C, and then annealed at 600°C for 2 h.

### Plasma Treatment of SnO_2_ Nanotube and Electrode (T-SnO_2_)

The obtained SnO_2_ nanotube was treated with Ar plasma at a flow rate of 30 ccm, powered at 100 V for 30 min. The glass substrate was treated with O plasma for 10 min to make the substrate more hydrophilic.

### Synthesis of SnO_2_@PANI on Electrode (PANI -T-SnO_2_)

28.6 ul aniline monomer was injected into 18 ml hydrochloric acid (1M), and then stirred for 30 min. After that, 0.0873 g of T-SnO2 was added and stirred for 30 minutes. Subsequently, 18 ml hydrochloric acid (1 M) containing 0.3 g ammonium persulfate (APS) was added in to the solution, and stirred for another 30 minutes. Finally, these two solutions were mixed and kept at a temperature of 0-4oC for 6 h in an ice bath. An Au-electrode was immersed for 2, 4, 8, and 10 minutes (denoted as 1-PANI-T-SnO2, 2-PANI-T-SnO2, 3-PANI-T-SnO2, 4-PANI-T-SnO2) in the solution until it turned dark blue, and then froze and dried it in a vacuum for 8 h.

## Material Characterization

The X-ray diffraction patterns (XRD) of serious samples were measured on the Dandong TD-3500 (Cu Kα radiation, 30 kV, 20 mA). The morphology was tested by the scanning electron microscope (SEM, Hitachi, SU-8010). The surface structure information was recorded with X-ray photoelectron spectroscopy (XPS) equipment (ESCA Lab MKII). The surface oxygen vacancy was characterized by Electron spin resonance (ESR) on Japanese electronics (JEOLFA200).

### Sensor Fabrication and Test

The gas sensing performances were tested as the same as our former work (Chen et al., 2020a), which were measured in an intelligent gas sensor analysis system (CGS-8, Beijing Elite Tech Co., Ltd.). To make sure the gas flow was smooth and steady, a self-made chamber (50 ml) and gas supply system were used. The test platform was shown in Figure 1B. All the tests were conducted at a room temperature of 25°C. The sensor response was defined as response = *R*g/*R*a, where *R*a is the resistance in air and *R*g is the measured resistance in the presence of the test gas.

## Results and Discussion

**Figure 2:**
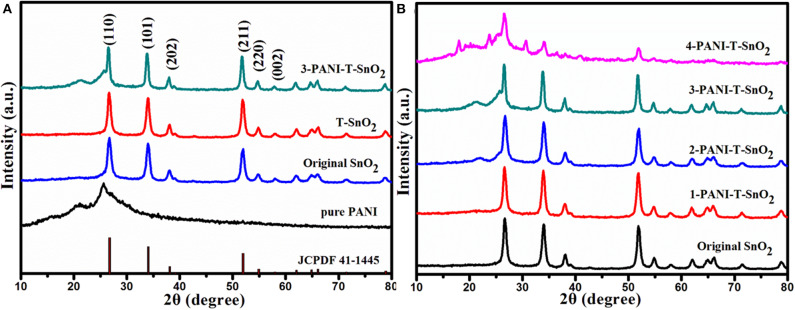
XRD pattern of different samples **(A)** different samples **(B)** SnO_2_ with different content of PANI. (T denoted as Ar plasma treatment, 1,2,3,4 denoted as different content of PANI).

**Figure 3:**
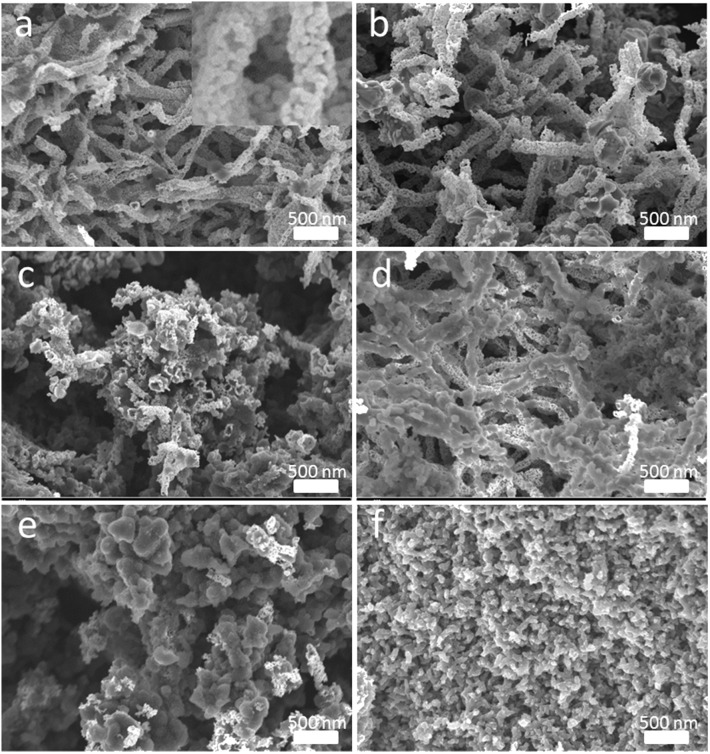
Morphology and surficial characteristics revealed by SEM images with different magnifications **(a)** Original SnO_2_, **(b)** T-SnO_2_, **(c)** 1-PANI-T-SnO_2_, **(d)** 2-PANI-T-SnO_2_, **(e)** 3-PANI-T-SnO_2_, **(f)** 4-PANI-T-SnO_2_.

The XRD pattern for different samples was shown in Figure 2. All the diffraction peaks could be ascribed to the rutile structure of SnO_2_ (JCPDS: 41-1445). As can be seen in Figure 2A, several characteristic peaks at 27.1°, 34.3°, 40.6°, and 52.1° should be ascribed to the crystal planes of (110), (101), (202), and (211) (Li et al., 2016; Su and Yang, 2016). No peak shift or new peaks could be found in the sample treated by Ar plasma, which implied that the Ar plasma treatment would not destroy the phase structure. The reason for this should be that the treatment only occurred on the surface and on a few destroyed atom layers, which would result in many surface defects like oxygen vacancies (Liu B. T. et al., 2018; Wu et al., 2018; Chen et al., 2020a). It was also found that there was little change on the diffraction peaks for the PANI coated samples, which should be due to the thinner PANI shell coating on the serious SnO_2_ samples. As the thickness of the PANI shell increased, the PANI peaks at 22.5^o^ and 26.5^o^ became much more obvious, as shown in Figure 2B. Obviously, these PANI shells encapsulate on the surface of SnO_2_ and could efficiently protect the surface defects. SEM was carried out as shown in Figure 3 for further research.

**Figure 4:**
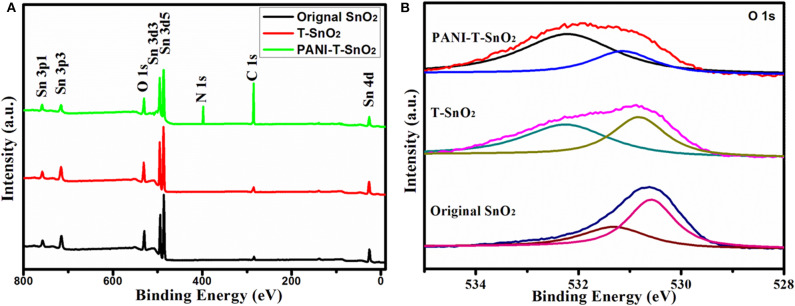
Full XPS spectral of different samples **(A)**; the high resolution XPS spectral of O1s **(B)**.

The SEM images of different SnO_2−_based samples were shown in Figure 3. Nanotube morphology, which was composited with many little grains, could be seen in the original SnO_2_ as shown in Figure 3a. The diameter of the nanotube was about 60–80 nm. Figure 3b showed the sample with Ar plasma treatment; no change could be found in it. This was consistent with the XRD results in Figure 2, which showed that the plasma could not affect the SnO_2_ crystalline structure. However, if the T-SnO_2_ sample was coated with the PANI shell, as shown in Figures 3c–f, the surface would become rough and irregular as the diameter increased. Additionally, if too many PANI were shown in Figure 3f, the morphology of the nanotube could not be found. XPS tests were performed in Figure 4 for detailed surface structure information.

The full XPS spectra of SnO_2_, T-SnO_2_, and PANI-T-SnO_2_ were list in Figure 4A. All the samples were composed of Sn, O, and C. However, the C peak in PANI-T-SnO_2_ was much higher than that in the other two uncoated samples, and there was also an N species peak, which implied that PANI is coated successfully. In addition, it was also found that the full XPS spectral of T-SnO_2_ seems to be unrelated with the original SnO_2_. For a deep investigation, the oxygen vacancies were further investigated by the O 1s spectra in Figure 4B. It could be clearly seen that the binding energy at 530.63 and 531.25 eV in the original SnO_2_ could represent the lattice oxygen in chemical groups Sn-O-Sn and adsorbed oxygen in Sn-OH and –OH, respectively (Li et al., 2016; Su and Yang, 2016). Compared with the original SnO_2_, the absorbed oxygen in O 1s curve for T-SnO_2_ and PANI-T-SnO_2_ exhibited a significant drift from 531.25 to 532 eV, which indicated the existence of oxygen vacancies (Chen et al., 2020a). Meanwhile, this peak in T-SnO_2_ was relatively lower than that in PANI-T-SnO_2_, which meant that some surface oxygen vacancies obtained by Ar plasma treatment in T-SnO_2_ would re-oxidate without any protective effect from the PANI shell. Obviously, it can be concluded that the PANI-T-SnO_2_ has a higher ratio and stable oxygen vacancies, which is beneficial for sensor properties.

**Figure 5:**
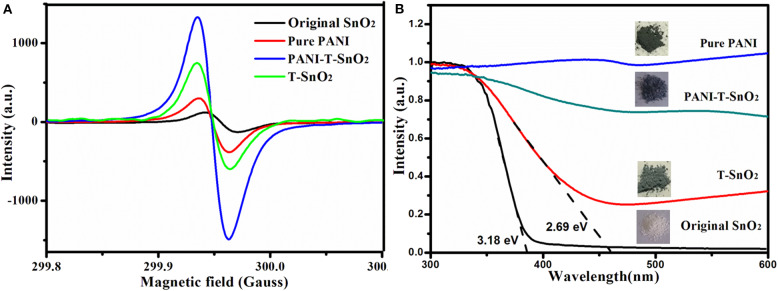
ESR curves of different processed samples **(A)**; absorption curve of different processed samples **(B)**.

An ESR test was used for a further study of the surface oxygen vacancies. It is generally known that the peak intensity at around g=2 in ESR curves usually represents the concentration of the surface oxygen vacancies (Xu et al., 2016; Chen et al., 2020a). That is to say, the Ar plasma treatment can result in many oxygen vacancies in T-SnO_2_, while the original SnO_2_ only contains limited defects (Xu et al., 2016; Chen et al., 2020a). Surprisingly, when T-SnO_2_ was coated with the PANI shell, the ESR signal of the surface oxygen vacancies was much higher than the uncoated samples. After considering that pure PANI only showed a weak signal, it could be concluded that this enhancement cannot be ascribed to the intrinsic properties of the coated PANI shell. The reason for this would be that the coated PANI shell can use air oxygen to protect the oxygen vacancies re-oxidization effectively. Of course, the relatively weak ESR signal in T-SnO_2_ should be ascribed to the fact that the test was not conducted in time, causing the re-oxidizing process to suffer (Chen et al., 2020a). The oxygen vacancies structure was also performed by absorption spectral as shown in Figure 5B. As shown, the absorption edge of the original SnO_2_ was 390 nm (3.18 eV), while it extended to 460 nm (2.69 eV) for T-SnO_2_. It is generally known that if the oxygen vacancies can broaden the absorption spectral, subsequently, the oxygen vacancies would affect its gas sensor properties (Wang et al., 2010; Xu et al., 2016; Cheng et al., 2017; Chen et al., 2020a), and for the sample coated PANI shell, the band gap would decrease further. This should be ascribed to the conductive properties of PANI, and implied that an effective hetero structure was constructed. It also showed that it can accelerate electron transfer during the sensing process (Chen et al., 2020a).

**Figure 6:**
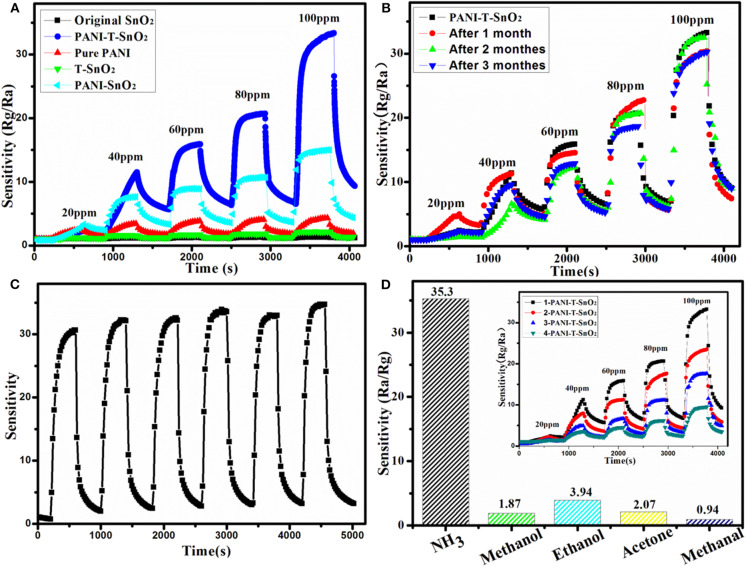
Resistance variation in gas sensing tests of different samples **(A)**, 3-monthes cycle stability test for PANI-T-SnO_2_ **(B)**, 100 ppm 6-time cycle test for PANI-T-SnO_2_ **(C)**, gas selectivity testing **(D)** (the inset is the gas sensing coated with different PANI layer).

The whole gas sensing test was carried out at 25°C with a relative constant humidity of 50%. In order to evaluate the response properties of different samples more intuitively, the gas response curves were shown in Figure 6A. As it shows clearly, the original SnO_2_ and T-SnO_2_ have nearly no sensitivity toward NH_3_ at room temperature and the pure PANI shows a relative lower sensitivity of about 4.5 at 100 ppm of NH_3_. However, the synergistic effect of surface oxygen vacancies and PANI coating could be observed, which could significantly enhance the sensitive response as we expected in PANI-T-SnO_2_. The result showed that the PANI-T-SnO_2_ has a higher response at 35.3 of 100 ppm, which was 7.8 times higher than pure PANI, and also better than most reported PANI-based materials as shown in Table 1. Obviously, the surface oxygen vacancies play the key role in the sensitive response that can rapidly separate and remove the surface electrons induced by the target gas (Chen et al., 2020a). An untreated sample coated with PANI was also listed for comparison; the response was about 15 at 100 ppm, which demonstrated our design concept that PANI can bring a room temperature response and the oxygen vacancies make it more sensitive, that is to say, that simply introducing surface defects or a coating of PANI it is not an efficient way to increase the gas sensor properties (Chen et al., 2020a). A 3-months multi-time stability gas response test and long cycle response performance were respectively listed in Figures 6B,C to investigate the stability of surface oxygen vacancies protected by the outer PANI layer. It showed that there were only a few tiny fluctuations for the sensor response after 3 months or after being retested six times, and remains at more than 90% sensitive at 100 ppm. This result further confirmed the PANI shell can protect the inner oxygen vacancies well and bring a stable gas response. There was nearly no change in the long cycle response performance under a concentration of 100 ppm. The response remained in a stable level, which illustrated a better stability, as we expected. In addition, this PANI shell had a thin layer which can exhibit a better sensor process as shown in the inset. And as shown in Figure 6D, there is substantially no response to the redox gas other than NH_3_, which shows an excellent selectivity for the target gas.

**Table 1:** Comparison of the performances of the sensor developed here and the other reported.

**Material**	**Gas**	**Temperature (**°**C)**	**The formula of response**	**Response**	**Concentration(ppm)**	**Reference**
Sb-doped SnO2	NH_3_	150	Rg/Ra	30	40 ppb	10.3
WO3@SnO2	NH_3_	200	Rg/Ra	25	50	13
RGO@SnO2	NH_3_	RT	Rg/Ra	22	100	10
RGO@SnO2	NH_3_	RT	Rg/Ra	1.3	100	11
MWCNT@SnO2	NH_3_	RT	Rg/Ra	5	100	12
PANI	NH_3_	RT	(Z-Z_0_)/Z	0.7	10	20
PANI	NH_3_	RT	Rg/Ra	2.2	10	21
PANI	NH_3_	RT	(Rg-Ra)/Ra (%)	7.90%	10	22
SnO_2_@PI	NH_3_	RT	Rg/Ra	33	100	18
SnO_2_@PANI	NH_3_	RT	Rg/Ra	25	80	27
CeO_2_@PANI	NH_3_	RT	(Rg-Ra)/Ra (%)	250%	50	29

**Figure 7:**
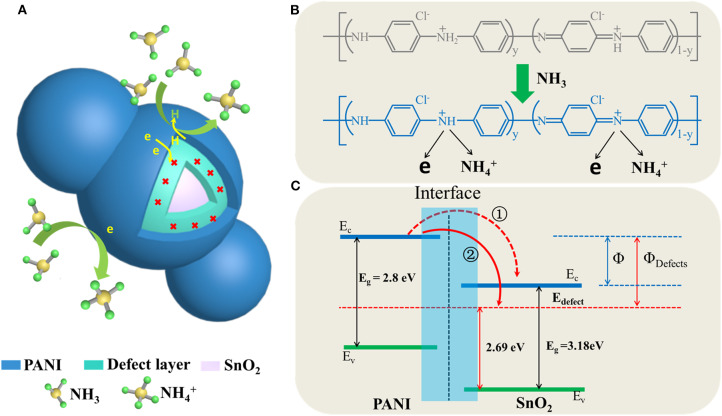
Schematic illustration of sensing mechanisms **(A)** schematic diagram of gas response of core-shell structure, **(B)** the interaction of NH_3_ molecules with the PANI, **(C)** heterojunction band structure and electron transfer diagram.

The schematic diagram of the detailed gas sensor mechanism was shown in Figure 7. It can be seen that the PANI shell acted as a better recognition center for electrons sensitive to NH_3_ since the SnO_2_nanotubes encapsulate with PANI, a heterojunction will was formed at their interface (Hu et al., 2002; Gumpu et al., 2014; Kumar et al., 2017; Chen et al., 2020a). If the PANI was active with HCl, it could efficiently generate more protons to gain conductive properties on the basis of a protonation reaction, as shown in Figure 7B. Thus, when it suffered a protonation reaction, it would form many more N^+^-H bonds, which would result in many positive centers, and then facilitate the free electrons in the valence band to move to these positive centers (Ikeda et al., 2012). The reaction process between the PANI shell and NH_3_ molecules was shown in Figure 7B, this chemisorption process is reversible, as equation (1) shows:

(1) $$\begin{array}{r} \text{PANI}-{\text{H}}^{+}+\text{N}{\text{H}}_{3}⇌\text{PANI}+\text{N}{\text{H}}_{4}^{+}+{\text{e}}^{-} \end{array}$$

The PANI shell would lose an H proton, and then recombine an electron from an H atom. With the reaction preceded, the electron generated by the PANI shell would gradually increase. Therefore, it would form a relatively high potential barrier, which would make the electron transfer more difficult, and then result in a lower response property. Thus, if the oxygen vacancies were introduced in the interface, it would recombine with the redundant electron, which could separate the generated electrons in PANI efficiently, and then accelerate the gas-sensitive reaction (Wang et al., 2017; Chen et al., 2020a). More importantly, the electron transfer ratio in Path 2 would be more efficient than that in Path 1, as the energy potential difference between the introduced oxygen vacancies energy level and the conduction band (CB) of PANI was relatively high, as shown in Figure 7C. It would also lead to higher sensitivity properties (Qin and Ye, 2016; Chen et al., 2020a).

## Conclusion

In conclusion, a PANI-T-SnO_2_ nanotube with rich and stable surface oxygen vacancies was constructed. The result shows that the surface oxygen vacancies act as an efficient electron acceptor, and generates more protons in the coated PNAI shell that react with the target NH_3_. It was found that the gas response of NH_3_ would be enhanced to 35.4 at 100 ppm at room temperature and concluded that the PNAI shell can also protect the oxygen vacancies from re-oxidation, which results in a stable and enhanced NH_3_ gas responsibility at room temperature. It can also lead to a stable response performance in multiple cycles of 3 months and multiple cycles.

## Author Contributions

All experimental work was performed by AJ under guidance of BL. HL, QL, and YY contributed to the analysis of the results and to the writing of the paper.

## Conflict of Interest

The authors declare that the research was conducted in the absence of any commercial or financial relationships that could be construed as a potential conflict of interest. The reviewer XY declared a shared affiliation, though no other collaboration, with the authors AJ, YY to the handling Editor.
